# Elective laparoscopic deroofing to treat the spontaneous rupture of a large simple liver cyst: a case report

**DOI:** 10.1186/s40792-016-0275-x

**Published:** 2016-12-07

**Authors:** Yuki Imaoka, Masahiro Ohira, Tsuyoshi Kobayashi, Seiichi Shimizu, Hiroyuki Tahara, Shintaro Kuroda, Kentaro Ide, Kohei Ishiyama, Hideki Ohdan

**Affiliations:** Department of Gastroenterological and Transplant Surgery, Applied Life Sciences, Institute of Biomedical and Health Sciences, Hiroshima University, 1-2-3 Kasumi, Minami-ku, Hiroshima, 734-8551 Japan

**Keywords:** Laparoscopy, Liver cyst, Rupture

## Abstract

**Background:**

The spontaneous rupture of nonparasitic liver cysts (NLC) is sometimes seen in clinical practice. However, there are no guidelines that describe the optimal treatment strategy and the surgical indications for an NLC rupture due to a small number of reports. Here, we present a case who underwent elective laparoscopic deroofing to treat a spontaneously ruptured NLC that had undergone conservative treatment.

**Case presentation:**

A 67-year-old woman was referred to our hospital for the evaluation of acute abdominal pain after the conservative treatment of an NLC at another hospital. She had stable vital signs and no abdominal rigidity. We performed an elective laparoscopic deroofing following an examination of the cyst relative to the bile ducts and the patient’s general condition. Computed tomography (CT) and magnetic resonance imaging (MRI) showed that there was no solid mass in the cyst. During the laparoscopic surgery, the cyst wall was resected and the back wall of the cyst was incinerated using an inverse-opal-structure electrode. The patient’s postoperative course was stable without any complications.

**Conclusions:**

We succeeded the conservative therapy and the elective laparoscopic surgery for ruptured of NLC. However, elective surgery in spontaneously ruptured NLC with intraabdominal infection or hemorrhage is still challenging.

## Background

Nonparasitic liver cysts (NLCs) are congenital benign malformations that occur in approximately 1–5% of the general population [1]. NLCs are usually asymptomatic, and they are found more frequently in women than in men at a ratio of 3:1 [1]. When NLCs reach substantial sizes, which occurs in 5% of the cases, they may become symptomatic and they can be associated with upper abdominal pain, bloating, nausea, vomiting, and dyspnea [2]. NLCs are commonly associated with a variety of complications, including hemorrhages, infections, and ruptures [3, 4]. The spontaneous rupture of NLC is sometimes seen in clinical practice. However, only 18 publications described NLC ruptures. Therefore, there are no guidelines that describe the optimal treatment strategy and the surgical indications for an NLC rupture [4–21]. Here, we describe a rare case of elective laparoscopic deroofing to treat an NLC that ruptured spontaneously after conservative treatment.

## Case presentation

**Fig. 1:**
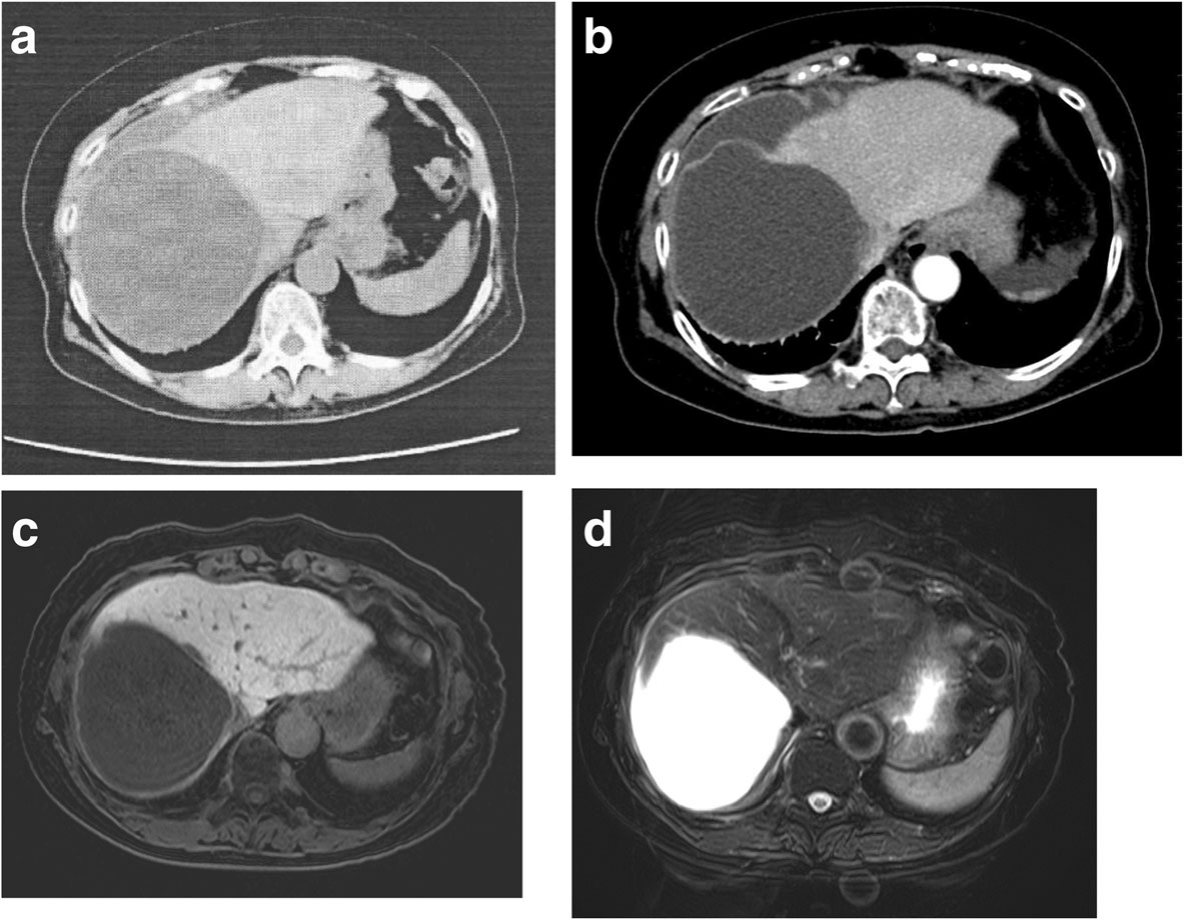
**a** Computed tomography (CT) scan before admission to hospital. **b** CT scan during the examination. **c** Magnetic resonance imaging (MRI) T1-weighted image. **d** MRI T2-weighted image

A 67-year-old Japanese woman was transferred to the emergency unit of our hospital for an evaluation of her acute abdominal pain. She had a 2-week history of conservative treatment with antibiotics for an NLC at another hospital (Fig. 1a).

On examination, the patient’s pulse rate was 80 beats/min, her blood pressure was 136/68 mmHg, and she did not have a fever. Her abdomen was flat but painful. The patient did not complain of nausea, vomiting, or diarrhea. She did not exhibit abdominal tenderness or muscular defense on her arrival at the clinic. The laboratory test results did not reveal liver dysfunction, and the patient’s total bilirubin level was 0.8 mg/dL, the prothrombin activity and international normalized ratio were 99% and 1.01, respectively, the creatinine level was 0.63 mg/dL, the platelet count was 313,000 /μL, and the albumin concentration was 3.9/dL. Other blood test results revealed acute inflammation, the absence of anemia, a white blood cell count of 16,350 cells/μL, a hemoglobin level of 13.4 g/dL, and a C-reactive protein level of 0.57 mg/dL. The levels of carcinoembryonic antigen and carbohydrate antigen 19-9 were within the normal ranges. There were no findings of neoplastic cysts in the abdominal ultrasound test. Enhanced computed tomography (CT) scanning showed a ruptured hepatic cyst without any extravasation, and a moderate amount of ascites fluid around the liver’s surface. The largest cyst was 10.5 cm and it was located on the anterior segment (Fig. 1b). The cyst’s volume had clearly declined compared with previous CT images. Magnetic resonance imaging (MRI) showed that the cyst did not contain a solid component (Fig. 1c, d). Based on the patient’s clinical course and the findings from the investigations, we determined that the ruptured NLC had not induced acute peritonitis; therefore, the patient received the antibiotics therapy (cefmetazole 3 g/day) prior to the surgery and was performed an elective laparoscopic deroofing after the patient’s general condition was assessed.

**Fig. 2:**
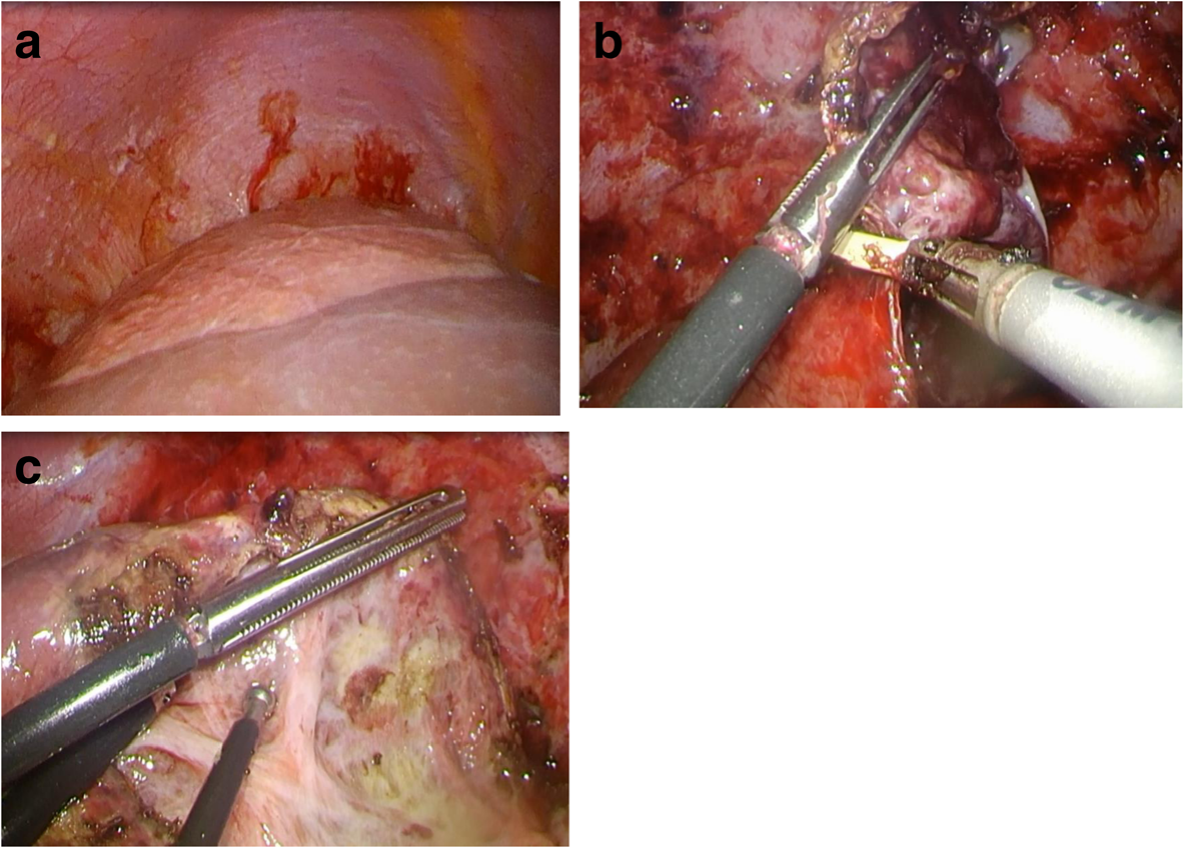
**a** The cyst on the right lobe. **b** The cyst wall was resected at the junction of the cyst. **c** The back wall of the cyst was incinerated using an inverse-opal-structure electrode while avoiding Glisson’s capsule

A laparoscopic deroofing was performed to rupture the NLC and achieve intraperitoneal drainage. Celioscopy was performed through a point below the umbilicus, and four trocars were necessary for the procedure. The 12-degree optical trocar was installed beneath the umbilicus with the 12-mm operator’s trocar in the epigastric region and two 5-mm trocars in the right hypochondrium and right subcostal region for apprehension. Harmonic^®^ shears (Ethicon US, LLC) and a bipolar cautery coagulation device were required. The cyst was on the right lobe and there were adhesions on the transverse colon, omentum, and peritoneum (Fig. 2a). The cyst was opened and 300 mL of brown and slightly muddled fluid were aspirated. No obvious hematomas, ascitic fluid, nodules, or other specific entities, for example, a malignant tumor, were detected. The culture of the peritoneal fluid showed no bile and no bacterium. The cyst wall was resected at the junction of the cyst and the liver parenchyma using an ultrasonic scalpel (Fig. 2b). The back wall of the cyst was checked carefully for evidence of a bile leak, and it was incinerated using an inverse-opal-structure electrode while avoiding Glisson’s capsule (Fig. 2c). The operation time was 133 min, and the total blood loss was 20 mL.

Pathological investigation revealed no evidence of malignancy and *Echinococcus* species infection. The patient’s postoperative course was stable and there were no complications. She was discharged on postoperative day 6. There have been no recurrences for 8 months.

## Conclusions

We have described a rare case of elective laparoscopic deroofing for the treatment of an NLC that ruptured spontaneously after conservative treatment. Since the rupture did not induce acute peritonitis and the patient’s vital signs were stable, the preoperative examination was carried out while the patient was being treated conservatively with antibiotics. We were able to avoid an emergency operation and to safely perform an elective laparoscopic deroofing without an NLC recurrence.

In general, ruptures of parasitic liver cysts tend to be caused by *Echinococcus* species, they are known complications associated with these cysts, and they are reported as hydatid cyst ruptures [5, 6]. On the other hand, a search of English language papers published in PubMed from 1959 to 2015 identified only 18 publications that describe NLC ruptures [4–21] (Table 1), and, of these, only one other case was treated using laparoscopy [14] (Table 2). Seven cases required emergency operations, and one case died postoperatively. Six of seven cases with intracystic bleeding required the emergency surgery. Intracystic bleeding would increase the tension inside the cyst and lead to rupture with shock. The other case has undergone left trisegmentectomy of the liver under a suspected diagnosis of cystadenocarcinoma as elective surgery [19]. Conservative management involving percutaneous drainage and antibiotics might be useful for cases who do not have peritoneal irritation and shock [4]. However, high recurrence rates have been reported after conservative treatment alone [22, 23]. Transcatheter arterial embolization (TAE) was reported as a useful option for spontaneous rupture of the cyst with intracystic bleeding and stable vital sign [17]. Unfortunately, the cyst had increased in size in a short period and required the simple cystectomy. More radical approaches, including cyst resections, atypical resections, and lobectomies, have been undertaken, and the recurrence rates were zero. However, these approaches are often associated with higher morbidity rates [24]. In relation to curability, the risk of relapse, and the possibility of other complications, including hemorrhages, cyst deroofing might be more favorable for most cases [2]. More recently, a laparoscopic approach was proposed and it was considered safe essentially [14, 25]. Increasingly, laparoscopic deroofing is being used in elective operations to manage NLCs that have not ruptured. The findings from a variety of studies have shown that laparoscopic deroofing is associated with reductions in morbidity, shorter hospital stays, and more rapid returns to normal activities, compared with open deroofing [26]. On the other hand, laparoscopic surgery in spontaneously ruptured NLC with intraabdominal infection or hemorrhage is still challenging. The exposure of the cyst may become difficult because the partially collapsed cyst would make the line of resection less obvious on laparoscopic examination [14]. And the active bleeding would make the views worse. In the cases of the intraabdominal infection such as acute peritonitis, the laparotomy surgery with drainage should be selected. Emergency surgery should be avoided, if possible, because they increase postoperative morbidity and mortality [27]. However, we should not hesitate to perform the emergency surgery. The ascites puncture should be the most reliable method to distinguish intraabdominal infection or hemorrhage.

**Table 1:** Literature review of ruptured nonparasitic liver cysts

Year	Reference	Age (years)	Sex	Symptoms	Peritoneal irritation	Cyst size (cm)	Location	Ascites	Properties of the ascites	Emergency procedures	Intracystic bleeding	Treatment	Outcome
2016	Imaoka et al.	67	F	Abdominal pain	No	10.5	Right lobe	Yes	Brown and muddled	No	No	Laparoscopic deroofing	Uneventful
2015	Inoue et al. [21]	59	F	Abdominal pain	Yes	10	Left lobe	Yes	Brown and muddled	Yes	Yes	Laparotomy and cyst fenestration	Uneventful
2013	Marion et al. [20]	37	F	Abdominal pain, dyspnea	No	18	Right lobe	Yes	Blood stained	Yes	Yes	Cystectomy	Uneventful
2010	Ueda et al. [6]	64	F	Abdominal pain	No	10	Right lobe	Yes	Serous brown	No	No	Percutaneous aspiration, injection of minocycline hydrochloride	Uneventful
2010	Miliadis et al. [12]	70	M	Abdominal pain	Yes	13	Right lobe	Yes	Opaque-yellowish peritoneal fluid	Yes	–	Deroofing of the cyst, omentoplasty	Uneventful
2007	Salemis et al. [13]	50	M	Abdominal pain, vomiting	Yes	17	Left lobe	–	–	Yes	–	Wide excision of the cyst, running locking suture along the edge of the resected cyst wall	Uneventful
2005	Cheung et al. [14]	73	F	Abdominal pain	Yes	17	Right lobe	Yes	Blood stained	Yes	Yes	Laparoscopic deroofing	Uneventful
2003	Shutsha and Brenard [15]	67	F	Abdominal pain	No	–	Multiple	Yes	–	No	No	conservative therapy	Uneventful
2003	Kanazawa et al. [16]	78	M	Abdominal pain	No	–	Right lobe	Yes	Blood stained	No	Yes	Conservative therapy	Uneventful
2002	Ishikawa et al. [17]	42	F	Discomfort in upper abdomen	No	10	S4/5	Yes	Brown and muddled	No	Yes	TAE, drainage, alcohol injection	Uneventful
2002	Carels and van Bommel [18]	76	M	Abdominal pain	Yes	19	Right lobe	Yes	Blood stained	Yes	Yes	Omentum placed over the ruptured cyst	Death 4 weeks after admission
1999	Yamaguchi et al. [19]	61	M	Abdominal pain	Yes	13	Left lobe	Yes	Blood stained	No	Yes	Hepatectomy	Uneventful
1999	Payatakes et al. [8]	62	–	Abdominal pain	–	9.5	Right lobe	–	–	–	–	Partial excision, external drainage	Uneventful
1989	Akriviadis et al. [4]	48	F	Abdominal pain	–	–	Left lobe	–	–	No	–	Conservative therapy	Uneventful
1988	Ayyash and Haddad [9]	36	M	Abdominal pain, vomiting	–	4	Right lobe	–	–	No	–	Conservative therapy	Uneventful
1974	Brunes [10]	54	F	Abdominal pain	–	25	Left lobe	–	–	–	–	Partial removal of the ruptured cyst	Uneventful
1972	Russell [11]	68	M	Abdominal pain	–	12	Left lobe	–	–	–	–	Left lobectomy	Uneventful
1960	Johnston [5]	82	F	Abdominal pain, vomiting	–	15	Right lobe	–	–	–	–	Drainage	Death 3 days after admission
1959	Morgenstern [7]	56	F	Abdominal pain	Yes	35	Left lobe	Yes	Brown	Yes	–	Lobectomy	Uneventful

*M* male, *F* female, *TAE* transcatheter arterial embolization

**Table 2:** Reports describing ruptured nonparasitic liver cysts treated with laparoscopy

Year	Reference	Age (years)	Sex	Symptoms	Peritoneal irritation	Cyst size (cm)	Location	Ascites	Properties of ascites	Emergency procedures	Intracystic bleeding	Treatment	Outcome
2016	Imaoka et al.	67	F	Abdominal pain	No	10.5	Right lobe	Yes	Brown and muddled	No	No	Laparoscopic deroofing	Uneventful
2005	Cheung et al. [14]	73	F	Abdominal pain	Yes	17	Right lobe	Yes	Blood strained	Yes	Yes	Laparoscopic deroofing	Uneventful

*F* female

Lai et al. [28] reported that the presence of biliary communication and malignancy could not be accurately determined preoperatively, despite technological advances. We suggest that careful examination of the cyst cavity at surgery remains the most reliable guide and leads to prevent intraoperative injury of biliary duct. The usefulness of an argon beam coagulation for preventing cyst recurrence was reported in 2003 [29]. We perform the coagulation of the remnant cyst wall as a routine technique. Another risk is the use of argon beam coagulation and electrocoagulation during surgery that could destroy a bile duct that is adjacent to the cyst’s wall, leading to postoperative bile leakage. The argon beam coagulator is also known to have a risk to the gas emboli due to increased intraabdominal pressure under laparoscopic condition. Analysis of the literature and experienced surgeons propose its use respecting some rules: avoiding direct application close to the parenchymal surface, no pulverization on small hepatic veins holes, and venting the abdomen (open trocars) in order to decrease the intraperitoneal pressure [30]. Nowadays, PDE camera using ICG was reported that it is important to detect bile duct and intraoperatively [31, 32].

Before this case, we had not treated a ruptured NLC at our hospital. Between 2006 and 2015, we undertook nine nonemergency laparoscopic deroofing for NLCs (Table 3). One case was transferred to undergo laparotomy, and none of the cases experienced complications. There were no differences between the present case and the other nine cases with respect to the operative findings and the postoperative outcomes, despite the current case experiencing a cyst rupture. Five cases had undergone imaging using DIC-CT, MRI, and magnetic resonance cholangiopancreatography (MRCP) to determine the courses that the bile ducts followed. The ascites fluid has a high signal and MRCP cannot provide the precious information in the cases with ascites. Therefore, MRCP was avoided in the present case.

**Table 3:** Cases with nonparasitic liver cysts treated with laparoscopy

Year	Age (years)	Sex	Location	Imaging	Treatment	Operation time (min)	Blood loss (mL)	Cyst size (cm)	Drainage volume (mL)	Complications	Hospital stay (days)
2006	80	F	Right lobe S5.6	DIC-CT	Laparoscopy	105	10	9	–	No	12
2006	14	F	Right lobe S7	–	Laparoscopy	155	20	18	–	No	5
2008	39	F	Right lobe S6	–	Laparoscopy	106	10	16	2800	No	10
2010	63	F	Multiple S2.3.7, S6	MRI	Laparoscopy → laparotomy	198	50	10	–	No	8
2010	77	F	Right lobe S7	–	Laparoscopy	135	20	18	3000	No	7
2011	66	F	Right lobe S5	–	Laparoscopy	129	10	12	–	No	4
2012	81	F	Multiple S8・S2	DIC-CT MRI	Laparoscopy	160	30	12	–	No	19
2013	78	F	Right lobe S6	MRCP	Laparoscopy	149	10	21	2800	No	6
2013	74	M	Right lobe S6	DIC-CT MRCP	Laparoscopy	226	190	24	5000	No	8
Average	63.6					151	39	16	3400	0/9	9
2016	67	F	Right lobe	MRI	Laparoscopy	133	20	10.5	300	No	6

*F* female, *M* male, *DIC-CT* drip infusion cholangiography computed tomography, *MRI* magnetic resonance imaging, *MRCP* magnetic resonance cholangiopancreatography

In conclusion, we succeeded the conservative therapy and the elective laparoscopic surgery for ruptured NLC. We suggest that the elective laparoscopic surgery is just one option, but is useful for the stable patients. However, elective surgery in spontaneously ruptured NLC with intraabdominal infection or hemorrhage is still challenging.

## Abbreviations

CT: Computed tomography
DIC-CT: Drip infusion cholangiography computed tomography
MRCP: Magnetic resonance cholangiopancreatography
MRI: Magnetic resonance imaging
NLCs: Nonparasitic liver cysts
TAE: Transcatheter arterial embolization
